# Neurologic Sequelae After Encephalitis Associated With Dengue Virus in Children

**DOI:** 10.1093/ofid/ofaf521

**Published:** 2025-09-10

**Authors:** Neha Srivastava, Rakesh Mankal, Rohit Beniwal, Aman Agarwal, Umaer Alam, Ashok Kumar Pandey, Rajni Kant, Mahima Mittal

**Affiliations:** ICMR–Regional Medical Research Centre, Gorakhpur, India; Department of Neurology, Baba Raghav Das Medical College, Gorakhpur, India; ICMR–Regional Medical Research Centre, Gorakhpur, India; ICMR–Regional Medical Research Centre, Gorakhpur, India; ICMR–Regional Medical Research Centre, Gorakhpur, India; ICMR–Regional Medical Research Centre, Gorakhpur, India; ICMR–Regional Medical Research Centre, Gorakhpur, India; Department of Pediatrics, All India Institute of Medical Sciences, Gorakhpur, India

**Keywords:** dengue, disability, encephalitis, outcome, sequelae

## Abstract

**Background:**

Neurologic complications associated with dengue infection have been increasingly recognized globally, particularly following detection of dengue virus in cerebrospinal fluid via polymerase chain reaction. Despite this, no prior study has assessed neurologic sequelae in patients with dengue-associated acute encephalitis syndrome (DEN-AES). This study aimed to evaluate the extent and domains of neurologic sequelae in pediatric DEN-AES cases.

**Methods:**

The study was conducted in 2023, including diagnosed DEN-AES cases (≤18 years) discharged between January 2018 and December 2019. DEN-AES was defined as acute fever onset with altered mental status (confusion, disorientation, coma, or inability to speak) and/or new seizures (excluding simple febrile seizures), as confirmed by NS1/IgM enzyme-linked immunosorbent assay and reverse transcription polymerase chain reaction tests for dengue. Long-term sequelae were assessed via home visits with a standardized disability assessment tool. Hospital records provided clinical, biochemical, and laboratory data for analysis.

**Results:**

Of 56 children (median age, 7.5 years [IQR, 5–10]; 53.6% male), neurologic sequelae of varying severity were observed in 22 (39.3%) cases. Severe disabilities were noted in 6 children, with 1 postdischarge death. Thirty-four children were fully recovered, scoring 5 on the Liverpool Outcome Score. Cognitive and behavioral impairments were the most common sequelae (37.5%), and 5 children required antiepileptic drugs for ongoing seizure management.

**Conclusions:**

Neurologic sequelae were identified in 39% of pediatric DEN-AES cases, underscoring the need for early diagnosis, continuous follow-up, and dedicated rehabilitation policies in dengue-endemic regions to support affected children.

## Teaser

A study on pediatric dengue-associated acute encephalitis syndrome in Eastern Uttar Pradesh found long-term neurologic sequelae in two-fifths of cases, including severe disabilities (10.7%) and cognitive/behavioral impairments (37.5%). Full recovery occurred in 60.7% of patients.

Dengue is a mosquito-borne viral infection caused by dengue virus (DENV), which belongs to the Flaviviridae family and consists of 4 distinct serotypes: DENV-1, DENV-2, DENV-3, and DENV-4 [1]. It is evident that infection with a specific serotype of DENV provides long-term immunity against that particular serotype, but subsequent infection with another serotype increases the risk of severe dengue [2]. Dengvaxia (Sanofi Pasteur) and TAK-003 (Qdenga; Takeda) live attenuated tetravalent vaccines are available for use in endemic countries [3].

About 14.6 million dengue cases and 12000 deaths have been reported globally in 2024 [4]. India, classified as group A for endemicity by the World Health Organization, also saw an increase in dengue cases in 2024 as compared with 2023 [4].

Initially, DENV was considered nonneurotropic, but later it was found to be neurovirulent when detected in cerebrospinal fluid by polymerase chain reaction testing [5]. Since then, the rising incidences of dengue-associated neurologic complications, including encephalitis, meningitis, myelitis, and myositis, are concerning [6–8]. While studies have linked dengue fever with encephalitis [6–8], the knowledge on neurologic sequelae after dengue-associated acute encephalitis is limited.

Eastern Uttar Pradesh of India has been endemic for seasonal outbreak of acute encephalitis syndrome (AES) since 1978, and children aged <15 years are the primary patients. Dengue contributes to 6% to 7% of total AES cases in the region [9]. DENV-2 is the major contributor of dengue-associated hospitalization in this region [10].

**Figure 1.**
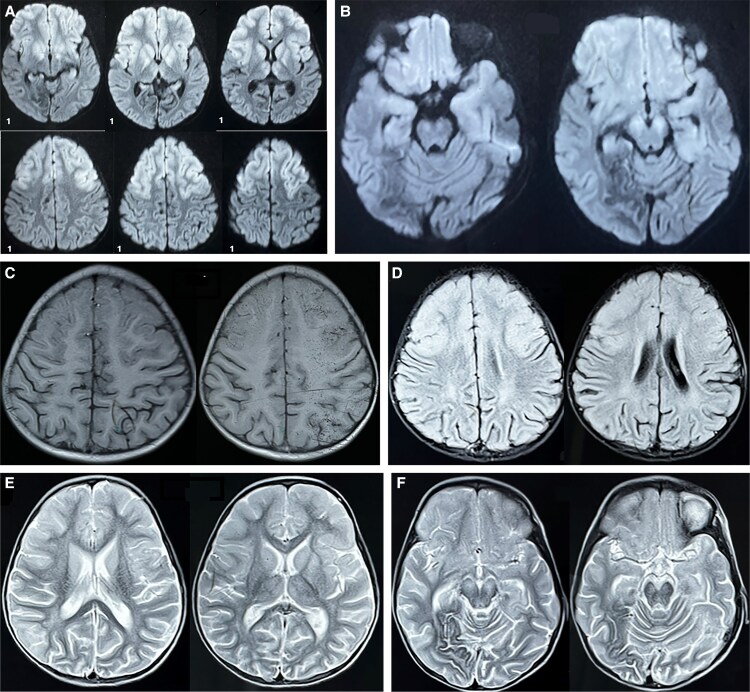
(*A, B*) Diffusion-weighted images show restriction in bilateral frontal cortex insula with bilateral basal ganglia, thalamus, and midbrain. (*C*) T1 image indicates hyperintensities in the bilateral frontal cortex. (*D–F*) T2/FLAIR images reveal white matter hyperintensities in bilateral frontal lobes with parietal and occipital lobes. Hyperintensities are also noted in bilateral basal ganglia, thalamus, and midbrain.

**Figure 2.**
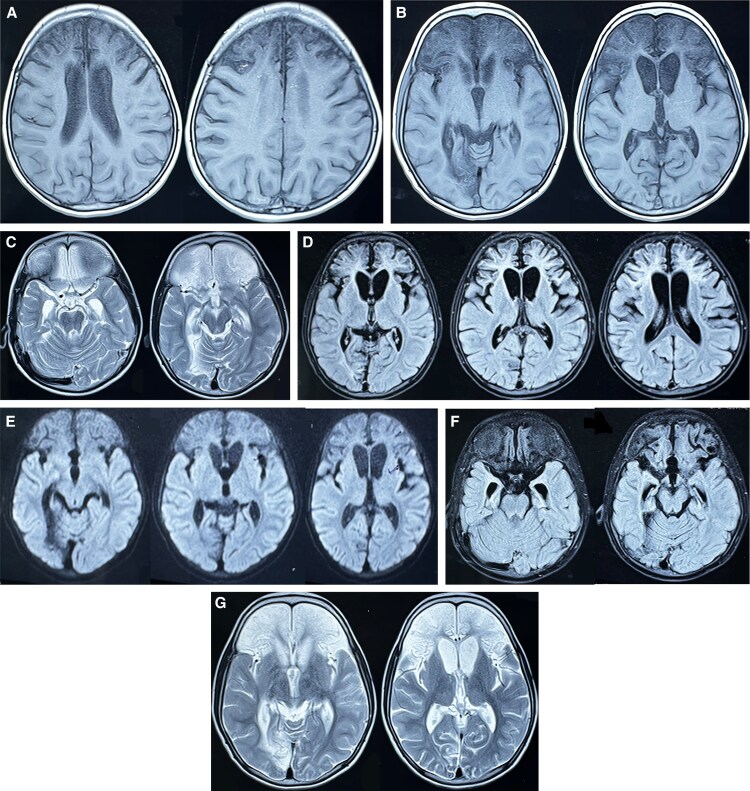
(*A,B*) T1 images show hypointensities with cortical atrophy in the bilateral frontal and occipital lobes with ex vacuo dilation of ventricles, including the prominence of ventricles. (C–G) T2/FLAIR images indicate encephalomalacic changes with surrounding gliosis in the bilateral frontal and right occipital lobes as sequelae of an old insult.

This retrospective cohort study was initiated in 2023 following the presentation of a 6-year-old female child at a tertiary care center in Gorakhpur with significant neurologic sequelae after recovery from AES in 2019. The child had been hospitalized in 2019 (age, 3 years old) with high-grade fever, seizures, and altered sensorium and was confirmed to have dengue-associated encephalitis by enzyme-linked immunosorbent assay (ELISA) and reverse transcription polymerase chain reaction. Despite treatment, she developed neurologic sequelae, including limb weakness, aphasia, and cognitive impairment. Neuroimaging conducted during the acute phase (Figure 1*A–F*) and follow-up (Figure 2*A–G*) revealed encephalomalacic changes and associated sequelae, confirming structural brain damage due to encephalitis. This case served as the impetus for the present study aimed at elucidating the clinical and functional outcomes of dengue-associated AES.

The current study was done to assess the extent and domains of long-term neurologic sequelae among pediatric patients with acute encephalitis diagnosed with dengue.

## METHODS

For this study, we identified patients using the AES line list maintained by the ICMR–Regional Medical Research Centre, Gorakhpur, per the AES management guidelines of the National Centre for Vector Borne Disease Control. This line list contains demographic and etiologic diagnosis details of all patients with AES hospitalized at Baba Raghav Das Medical College, Gorakhpur.

We screened pediatric cases (≤18 years of age) from the 2018 and 2019 line lists. Only patients who tested positive for DENV and negative for all other AES etiologies were included. We then retrieved the clinical, biochemical, and laboratory investigation details of these patients with dengue-positive AES from the hospital records. Neuroimaging was not routinely performed for all encephalitis cases due to limited availability of imaging resources and clinical constraints.

### Patient Consent Statement

Ethical approval from the Institutional Human Ethical Committee of the ICMR–Regional Medical Research Centre (EC No. RMRCGKP/EC/2023/3.16) was obtained to follow up on all patients aged ≤18 years who were hospitalized with acute encephalitis, had confirmed DENV, and were discharged from January 2018 to December 2019. Written informed consent and assent were obtained from the parents, legal guardians, or caregivers of the children during follow-up by home visit to ensure their participation. The study adhered to the STROBE reporting guidelines.

Demographic, clinical, and biochemical data were collected from the hospital records of pediatric patients (≤18 years) diagnosed with AES and hospitalized in the encephalitis ward of Baba Raghav Das Medical College from January 2018 to December 2019. An AES case was defined “a person of any age, at any time of the year, characterized by the sudden onset of fever and altered sensorium or altered mental status (such as confusion, disorientation, coma, or inability to speak) and/or the occurrence of new seizures (excluding simple febrile seizures).” The etiologic diagnosis of AES cases was done per the AES testing protocol (Supplementary File 1). AES cases were included if they tested positive for dengue NS1 and IgM in serum by ELISA (Supplementary File 1). The Liverpool Outcome Score (LOS) [11] was used to evaluate disability during follow-up (Supplementary File 2).

The LOS is a validated questionnaire tool for evaluating functional disability in children following encephalitis. The LOS consists of 5 graded categories: 5, full recovery; 4, minor sequelae not interfering with daily activities; 3, moderate disability with some interference in daily function; 2, severe disability requiring significant assistance; and 1, death. For the purpose of this study, outcomes were dichotomized into good (LOS 5) and poor (LOS 1–4). Patients classified with LOS ≤2 were considered to have severe disability (Supplementary File 2).

For statistical analysis, dengue-associated encephalitis cases fully recovered at the time of follow-up (LOS 5) were categorized as having a good outcome, while those that did not recover (LOS 1–4) were categorized as having a poor outcome (died or had disability or sequelae at follow-up). Demographic, clinical, biochemical, and laboratory parameters collected at the time of admission to the tertiary care center were compared between these groups. Continuous variables such as age, blood pressure, and laboratory values (eg, Glasgow Coma Score, serum sodium, platelet count) were analyzed by the independent samples *t* test. This test was employed to compare the means between 2 independent groups (good vs poor outcome) and assess whether there were any statistically significant differences. Results were presented as mean and SD. For categorical variables such as age groups, gender, and clinical symptoms, the χ^2^ test or Fisher exact test was used to compare the proportions between the outcome groups. Statistical significance was defined as *P* < .05.

Patients aged >18 years, those with missing contact information, those who did not provide consent, and those who were absent 3 times during follow-up visits were excluded. Additionally, as a potential exclusion criterion, patients with neurologic sequelae were asked if they had experienced any accidents or required further hospitalizations (unrelated to AES) after being discharged from the hospital, although no such case was found in our study.

## RESULTS

A total of 56 dengue-positive AES cases (55 by NS1 ELISA and 1 by IgM ELISA) were discharged from the pediatric ward of Baba Raghav Das Medical College from January 2018 to December 2019. The 3-year-old child mentioned in the case history (6 years old at the time of the study) is 1 of the 56 patients included here. All 56 were reachable for a home visit and agreed to participate in the study. The LOS and its interpretation are detailed in Table 1. Disability of varying severity levels was observed in 39.3% of patients (n = 22). One patient experienced posthospitalization death, while severe disability was observed in 6 patients (10.7%). Full recovery occurred in 60.7% of patients. Cognitive and behavioral impairments were prevalent among the majority of patients. Additionally, 5 patients were experiencing seizures and were taking antiepileptic drugs at the time of follow-up.

**Table 1.** Extent of Poor Outcome and Domains of Neurologic Sequalae Among Pediatric Cases of Dengue-Associated Encephalitis (N = 56)

LOS^a^	Patients, No. (%)	Interpretation
1	1 (1.8)	Death
2	6 (10.7)	Severe sequalae: impairing function sufficient to make patient dependent
3	4 (7.1)	Moderate sequalae: mildly affecting function, probably compatible with independent living
4	11 (19.6)	Mild sequalae: having a minor effect on physical function, causing a personality change, or requiring medication
5	34 (60.7)	Fully recovered

Domains of Sequelae^b^		Parameters in domain
Cognition and behavior	21 (37.5)	Speech/communication, hearing, behavior, recognition, bladder and bowel control
Mobility	3 (5.4)	Sitting, standing up, walking, hands on head, picking up
Self-care	6 (10.7)	Feeding/eating, leaving alone, dressing, bladder and bowel
Life activities	10 (17.9)	School and working

Abbreviation: LOS, Liverpool Outcome Score.

^a^For analysis, 15 questions/activities of the LOS were grouped into 4 domains of disability (cognition and behavior, mobility, self-care, and life activities).

^b^Patients may have had more than 1 type of disability. Each patient is represented in all applicable disability categories.

Movement disorders were observed in our cohort, with dystonia present in 4 patients, chorea in 1, and tics in 1. Clinical characteristics and laboratory parameters compared between the poor and good outcome groups (Table 2) showed that vomiting occurred more frequently in the poor outcome group than the good outcome group. Laboratory findings indicated significantly lower mean platelet counts in the poor outcome group than the good outcome group. Total mean bilirubin levels were also higher in the poor outcome group vs the good outcome group. Additionally, cerebrospinal fluid protein levels were significantly elevated in patients with poor outcomes as compared with those with good outcomes. There were no differences in neurologic presentation between the outcome groups.

**Table 2.** Baseline Characteristics of Dengue-Associated Encephalitis Cases for Outcome

	Mean ± SD or No. (%)		
Characteristic	Good Outcome (n = 34)	Poor Outcome (n = 22)	*P* Value
Age, y	8 ± 3.8	6.7 ± 3	.116
Age group, y			.157
0–5	10 (34.3)	7 (22.3)	
6–11	16 (47)	14 (63.6)	
12–18	8 (23.5)	1 (4.5)	
Gender			.666
Female	15 (44.1)	11 (50)	
Male	19 (55.6)	11 (50)	
Clinical signs and symptoms			
Fever onset, d	7.5 ± 6	7.6 ± 4.7	.983
Multiple seizures (>2 episodes in last 24 h)	19 (55.6)	13 (59)	.813
Multiple episodes of vomiting (>3 episodes in last 24 h)	12 (35.3)	13 (59)	.080
Altered sensorium^a^	26 (76.5)	15 (68.2)	.494
Headache	6 (17.6)	3 (13.6)	.690
Abdominal pain	10 (29.4)	7 (31.8)	.848
Total Glasgow Coma Scale	9 ± 3	9 ± 3.6	.984
Blood pressure, mm Hg			
Systolic	88.8 ± 14.1	87.8 ± 10.2	.783
Diastolic	53.1 ± 14.3	55.7 ± 7.8	.453
Total leucocyte count/mm^3^	21 119.35 ± 25 124.12	13 795.24 ± 6984.66	.200
Hemoglobin, mg/dL	9.1 ± 2.3	9.7 ± 2.5	.407
Platelet count, 10^3^/mm^3^	176 047.6 ± 125 586.8	106 093.8 ± 59 899.57	.008^b^
Serum			
Sodium	63.65 ± 68.6	48.9 ± 66.4	.431
Potassium	1.97 ± 2.2	1.88 ± 2.6	.892
Glutamic oxaloacetic transaminase, IU/L	157.8 ± 107.4	151.6 ± 159.8	.867
Glutamate pyruvate transaminase, IU/L	108 ± 77.8	119 ± 150	.740
Total bilirubin, mg/dL	0.6 ± 0.4	1.1 ± 1	.048^b^
Serum creatinine, mg/dL	0.7 ± 0.3	0.7 ± 0.4	.704
Cerebrospinal fluid, mg/dL			
Protein	99.4 ± 47.7	143.7 ± 77.3	.023^b^
Glucose	57.9 ± 27.6	54.1 ± 27.5	.616

^a^Altered sensorium was defined as impaired consciousness (confusion, disorientation, stupor, or coma) lasting ≥24 hours, consistent with diagnostic criteria for encephalitis.

^b^ *P* < .05.

## DISCUSSION

This cohort study highlights the significant long-term neurologic sequelae observed in approximately 40% of pediatric dengue-associated encephalitis cases. While neurologic disorders in dengue fever have been well documented [5–8, 12–14], knowledge on its long-term impact on neurologic functioning is limited. To our knowledge, our study is the first to assess long-term neurologic sequelae among pediatric cases of dengue-associated encephalitis and various domains of neurologic impairment.

An increase in reports of DENV invasion into the central nervous system has broadened its clinical spectrum [15–17]. DENV-2 and DENV-3 are most commonly found in AES cases [15]. Among all central nervous system infections, DENV now accounts for approximately 4% to 13% of total cases [16, 17]. Previous studies have identified common movement disorders linked to dengue infection, such as dystonia, parkinsonism, opsoclonus-myoclonus syndrome, and ataxia [18–20]. Ataxia has been reported as the most frequently encountered movement disorder in previous studies [20]. However, in our cohort, dystonia was the predominant movement disorder. Case reports on dengue-associated encephalitis often show involvement of the basal ganglia, thalamus, temporal lobes, hippocampus, cerebellum, and cerebral white matter [21], similar to common imaging features seen in Japanese encephalitis, chikungunya encephalitis, and herpes encephalitis [22, 23]. In our index case, magnetic resonance imaging findings during the acute phase revealed hyperintensities on T2 and FLAIR sequences involving bilateral frontotemporoparietal cortices, basal ganglia, thalamus, and midbrain, with restricted diffusion suggestive of encephalitic involvement (Figure 1*A–F*). On follow-up imaging performed 2 years later, there was evidence of significant cortical atrophy, encephalomalacic changes with surrounding gliosis in the bilateral frontal lobes and right occipital lobe, and ex vacuo dilatation of the lateral ventricles (Figure 2*A–G*), consistent with sequelae seen in postencephalitic conditions [24].

The strength of our study is that all assessment for neurologic sequelae was done by trained health care professional using a validated and standardized disability assessment tool. However, the retrospective nature and limited sample size in the study are the major limitations.

In conclusion, our study provides useful information on the long-term impact of dengue-associated encephalitis on the functional outcome and quality of life of pediatric dengue cases. Future studies should investigate factors that can predict disability in hospitalized patients with dengue, and they should develop a tailored rehabilitation or intervention package for individuals with disability. The extensive dengue vaccination program with available vaccines in endemic regions may prevent severe outcome in infected cases.

## Supplementary Material

ofaf521_Supplementary_Data

## Supplementary Data

Supplementary materials are available at *Open Forum Infectious Diseases* online. Consisting of data provided by the authors to benefit the reader, the posted materials are not copyedited and are the sole responsibility of the authors, so questions or comments should be addressed to the corresponding author.
