# Supplementary material for: Neurologic Sequelae After Encephalitis Associated With Dengue Virus in Children
Source: Open Forum Infect Dis. 2025 Sep 10;12(10):ofaf521. doi: 10.1093/ofid/ofaf521 (PMC12480735; doi:10.1093/ofid/ofaf521)
Supplement: ofaf521_Supplementary_Data [file ofaf521_supplementary_data.zip › Supplementary file 1.docx]

**Supplementary file 1**

**Diagnostic algorithm for diagnosing AES cases at ICMR-Regional Medical Research Centre, Gorakhpur**

| **Etiologies** | **Test** | **Samples** | **Kit** | **References** |
| --- | --- | --- | --- | --- |
| *Japanese encephalitis Virus* | IgM ELISA | Serum, CSF | JEV-specific IgM antibody capture ELISA (MAC-ELISA) | 1 |
| *Orientia tsutsugamushi* (Scrub typhus) | IgM ELISA | Serum, CSF | Scrub Typhus Detect IgM ELISA System (INBIOS International, Inc., USA) | 2 |
| *Dengue Virus* | NS1 and IgM ELISA | Serum | DEN NS1 antigen ELISA (J. Mitra and Co Pvt. Ltd, New Delhi, India)  Dengue IgM Capture ELISA kit (developed by ICMR-National Institute of Virology Pune) | 3,4 |
| Leptospira | IgM | Serum | PanBio Leptospira IgM; Alare, Australia | 5 |
| *Chikungunya Virus* | IgM | Serum | CHIK IgM Capture ELISA kit (developed by ICMR-National Institute of Virology Pune) | 6 |

CSF: Cerebrospinal fluid; ELISA: Enzyme linked Immunosorbent Assay;

1. World Health Organization. Manual for the Laboratory Diagnosis of Japanese Encephalitis Virus Infection. no. March, pp. 1–52, 2007.
2. Kamble S, Mane A, Sane S, et al. Seroprevalence & seroincidence of *Orientia tsutsugamushi* infection in Gorakhpur, Uttar Pradesh, India: A community-based serosurvey during lean (April-May) & epidemic (October-November) periods for acute encephalitis syndrome. *Indian J Med Res*. 2020;151(4):350-360. doi:10.4103/ijmr.IJMR_1330_18
3. Behera SP, Bhardwaj P, Deval H, et al. Co-circulation of all the four Dengue virus serotypes during 2018-2019: first report from Eastern Uttar Pradesh, India. *PeerJ*. 2023;11:e14504. Published 2023 Jan 9. doi:10.7717/peerj.14504
4. <https://www.icmr.gov.in/icmrobject/custom_data/1702883738_niv_den_kit.pdf>
5. Shukla S, Mittal V, Karoli R, Singh P, Singh A. Leptospirosis in central & eastern Uttar Pradesh, an underreported disease: A prospective cross-sectional study. *Indian J Med Res*. 2022;155(1):66-72. doi:10.4103/ijmr.IJMR_1811_19
6. <https://main.icmr.nic.in/sites/default/files/Technology%20for%20Collaboration/NIV_CHIK_kit.pdf>
