# Supplementary material for: Neurologic Sequelae After Encephalitis Associated With Dengue Virus in Children
Source: Open Forum Infect Dis. 2025 Sep 10;12(10):ofaf521. doi: 10.1093/ofid/ofaf521 (PMC12480735; doi:10.1093/ofid/ofaf521)
Supplement: ofaf521_Supplementary_Data [file ofaf521_supplementary_data.zip › Suppl_Liverpool_Outcome_Score_Children_Follow_Up.pdf]

Liverpool Outcome Score (LOS) for Assessing Children at <u>Follow-Up</u>

Name: ___________________________ Date of birth (dd/mm/yy): ___ ___ / ___ ___ /___ ___

Age: _______________ Study No. _____

Today's date (dd/mm/yy): ___ ___ /___ ___ /___ ___ Sex: Male ☐ Female ☐

Date of original admission to hospital (dd/mm/yy): ___ ___ /___ ___ /___ ___

Date discharged from hospital (dd/mm/yy): ___ ___ /___ ___ /___ ___

Relationship of person with child (e.g., mother/aunt): ____________________________

Name of person completing form – please print: ________________________________

Job Title: _________________________

Answer each question. <u>Circle or underline the correct answer, and write the score in the column</u>

<u>Ask the parent or caregiver the following questions:</u>
For some of these questions, you ask the parent or caregiver how this child compares with other children of a similar age in their locality, e.g., how does this child compare in speaking or walking or talking to other children of the same age n the community.

<u>1. Speech or communication</u>:
Compared with other children the same age in the community, is the child's speech or communication:

- The same as other children of this age (5)
- Changed or reduced (3)
- Not speaking or communicating (2) _______

<u>2. Feeding</u>
The child's feeding is:

- The same as other children (5)
- Occasionally needs help (3)
- Always needs more help (2) _______

<u>3. Leaving Alone</u>
Before the illness, could a child of this age be left alone without coming to harm?

- If **No** score 5 (5)

If **Yes**, can this child be left alone now?

- Yes (5)
- Yes briefly in familiar environment (3)
- No (2) _______

4. <u>Behaviour</u>
Compared with other children of this age, do the caregivers think the child's behaviour is altered?

- No, completely normal (5)
- Gets angry easily (4)
- Other behavioural problems (4)
- Severely abnormal (2) _______

If abnormal give details____

5. Recognition
Can other children of this age recognise their relatives, other than their main carer?
- If **No**, score 5 (5)

If **Yes,** can this child recognise their relatives, other than their main carer?
- Yes (5)
- Some (3)
- None (2) _______

6. School and working
Are other children of the same age at school or working?

If **Yes**, is the child
- Now back to normal at school or work (5)
- Not doing as well (4)
- Dropped a school grade or no longer attending school or work (3)

If **No**, is the child:
- Still able to do the same tasks at home, follow the same routine, or play normally? (5)
- Not able to do as well as before (4)
- Not able to do at all (3) _______

7. Epilepsy/ Seizures
Has the child had any seizures in the last 2 months?
- No seizures and not on anti-epileptic drugs (5)
- No seizures and on anti-epileptic drugs (4)
- Yes, has had seizures (3)
- Yes, seizures most days (2) _______

8. Dressing
Can other children of this age dress themselves?
- If **No**, score 5 (5)

If **Yes**, can this child dress themselves?
- Yes (5)
- Occasionally needs more help (3)
- Always needs more help than other children of the same age (2) _______

9. Bladder and Bowel control
Is urinary and faecal continence:
- The same as other children the same age (5)
- Occasionally needs more help or occasionally is incontinent (4)
- Needs more help or is incontinent of bowel or bladder (2) _______

10. Hearing
Does the parent think this child's hearing is:
- Normal (5)
- Reduced in one or both ears (4)
- Cannot hear at all (3) _______

Liverpool Outcome Score (LOS) for assessing Children at Follow-Up.

Observation of the child's abilities
For these questions you observe what the child can do. If you cannot get the child to cooperate, answer these questions based on what the caregiver says.

11. Sitting
Can other children of the same age sit?
- If **No,** score 5 (5) _______

If **Yes**, observe, can this child sit?
- Yes, independently (5)
- Needs help (3)
- Not at all (2) _______

12. Standing up
Can other children of this age get from sitting to standing?
- If **No**, score 5 (5) _______

If **Yes**, observe, can the child get from sitting to standing?
- Yes, independently (5)
- Needs help (3)
- Not at all (2) _______

13. Walking
Can other children of this age walk?
- If **No**, score 5 (5)

If **Yes**, observe this child walking 5 metres across room. The child walks:
- Normally (5)
- Abnormally, but independently +/- crutches/stick (3)
- Not able to walk (2) _______

14. Hands on head
Put both your hands on your head, and ask the child to copy you. Child is:
- Too young (5)
- Normal both hands (5)
- Abnormal one or both hands (4)
- Unable one or both hands (3) _______

15. Picking Up
Ask child to pick up pea-sized ball of paper or small coin:
- Normal pincer grasp both hands (5)
- Unable one hand (3)
- Abnormal one hand or both hands (3)
- Unable both hands (2) _______

Outcome Score =Lowest score for any single question (range 2-5) _______

Total Score = all the individual scores added up (range 33 –75) _______
(If the child died, the score = 1)

Any other Comments: ____

____

____

Liverpool Brain Infections Group
UNIVERSITY OF LIVERPOOL
Brain Infections Global

The Final Liverpool Outcome Score is the lowest number scored for any question single question
5 = Full recovery
4 = Minor sequelae with no effect, or only minor effects on physicial function; or personality change; or on medication.
3 = Moderate sequelae mildly affecting function, probably compatible with independent living
2 = Severe sequelae, impairing function sufficient to make patient dependent
1 = Death

Notes

The Liverpool Outcome Score (1) was developed by the University of Liverpool Brain Infections Group with the support of PATH and the Bill and Melinda Gates Foundation as part of the international Japanese encephalitis control consortium (2). It is available to be used freely by clinicians working on encephalitis and other brain conditions. It has seen been used to assess outcomes in a range of brain infections in adults and children. It is reproduced here through *Brain Infections Global* – a UK National Institute for Health Research (NIHR) Global Health Research Group on Acute Brain Infections run by Liverpool Brain Infections Group and partners. You can get more information on *Brain Infections Global*, including access to our other teaching resources, here: https://braininfectionsglobal.tghn.org/brain-infections-global-training/.

References

1. Lewthwaite P, et al. Disability after encephalitis: development and validation of a new outcome score. Bull World Health Organ 2010; 88: 584-92.

2. Solomon T. Control of Japanese encephalitis--within our grasp? New England Journal of Medicine 2006; 355: 869-71.

For an updated list of publications using the score, click here.
